# Association of ficolin single nucleotide polymorphism with systemic lupus erythematosus in the Chinese Han Population

**DOI:** 10.1515/rir-2025-0023

**Published:** 2025-10-04

**Authors:** Feng Yin, Jinhua Gu, Ping Zhao, Jie Chen

**Affiliations:** Department of Rheumatologyand Immunology, The Second People’s Hospital of Yibin, Yibin, Sichuan Province, China; Department of Rheumatology and Immunology, The Affiliated Hospital of Southwest Medical University, Luzhou, Sichuan Province, China

Dear Editor,

Systemic lupus erythematosus (SLE) is a heterogeneous incurable autoimmune disease and can involve one or more organs.^[1]^ The pathogenesis of SLE is still poorly understood. Studies have shown that SLE has a strong but incompletely understood genetic architecture.^[2]^ Genetic interactions with environmental factors might initiate the disease, resulting in immune dysregulation.^[3]^ Ficolins, encoded by FCN, belong to an important family of pattern recognition molecules and have crucial functions in the innate immune system.^[4]^ Three human ficolins have been identified, termed ficolin-1, ficolin-2, and ficolin-3. To date, FCN has been analyzed in a number of diseases. Addobbati Catarina revealed that the T/T genotype for FCN rs3124954 single nucleotide polymorphism (SNP) was related to the occurrence of nephritis.^[5]^ The *GG* genotype and *G* allele for FCN rs3124952 were significantly more represented in patients with Pediatric-onset SLE.^[6]^ Therefore, we hypothesized that FCN gene may be an important susceptibility gene for SLE.

A total of 146 SLE patients were recruited as the case group and 140 healthy individuals composed the control group. All subjects were the Chinese Han and were recruited from the First Affiliated Hospital of Southwest Medical University during the period from 2022.06 to 2022.12. SLE patients were fulfilled the 2019 American College of Rheumatology Criteria. Informed consent was obtained from all individuals before the study, which was approved by the Medical Ethics Committee. Selecting the three FCN gene SNPs was according to some previous studies about the relationship with SLE or other rheumatic disease, and/or their possible functional impact. We observed that there were no significant differences in allele frequency and genotype distribution of FCN s7851696, rs2989727 and rs1071583 between SLE cases and healthy individuals. The results of association analysis between mentioned SNPs and SLE were shown in Table 1. The distributions of alleles and genotypes of SNPs in SLE patients were shown in Table 2.

**Table 1:** Statisfied analysis of FCN gene SNPs and SLE susceptibility.

SNPs	Genotypes	Case (*n*,%)	Controls (*n*,%)	OR (95%CI)	*P* value
Rs2989727	CC	40(27%)	43(31%)	reference	0.344
	CT	79(54%)	77(55%)	0.365 (0.075,1.791)	0.214
	TT	27(19%)	20(14%)	0.389 (0.108,1.41)	0.151
Rs7851696	GG	90(62%)	83(59%)	reference	0.579
	TG	53(36%)	50(36%)	2.344 (0.472,11.65)	0.298
	TT	3(2%)	7(5%)	2.184 (0.433,11.015)	0.344
Rs1071583	CC	34(23%)	31(22%)	reference	0.422
	CT	80(55%)	74(54%)	0.41 (0.086,1.95)	0.236
	TT	32(22%)	35(24%)	0.878 (0,271,2.785)	0.826

**Table 2:** The distributions of alleles and genotypes of SNPs in SLE patients.

Clinical features	Rs2989727							Rs7851696							Rs1071583						
	Genotype (*n*)			*P*	Allele (*n*)		*P*	Genotype (*n*)			*P*	Allele (*n*)		*P*	Genotype (*n*)			*P*	Allele (*n*)		*P*
	CT	TT	CC	value	C	T	value	TG	TT	GG	value	G	T	value	CT	TT	CC	value	C	T	value
Anti-Sm																					
positive	38	18	21	0.249	80	74	0.364	27	2	48	0.945	123	31	0.973	40	17	20	0.688	80	74	0.648
negative	41	9	19		79	59		26	1	42		110	28		40	15	14		68	70	
Anti-dsDNA																					
positive	36	11	19	0.858	74	58	0.616	21	2	43	0.474	107	25	0.625	38	14	14	0.81	66	66	0.832
negative	43	16	21		85	75		32	1	47		126	34		42	18	20		82	78	
Anti-rRNP																					
positive	47	16	20	0.59	87	79	0.421	27	3	53	0.248	133	33	0.873	48	15	20	0.433	88	78	0.361
negative	32	11	20		72	54		26	0	37		100	26		32	17	14		60	66	
Arthritis																					
positive	41	18	22	0.41	85	77	0.448	26	2	53	0.505	132	30	0.423	42	17	22	0.465	86	76	0.36
negative	38	9	18		74	56		27	1	37		101	29		38	15	12		62	68	
Rash																					
positive	54	15	28	0.408	110	84	0.278	35	2	60	1.00	155	39	0.951	59	20	18	0.086	95	99	0.409
negative	25	12	12		49	49		18	1	30		78	20		21	12	16		53	45	
Alpecia																					
positive	23	8	12	0.998	47	39	0.97	11	1	32	0.133	75	13	0.129	24	9	11	0.932	46	42	0.722
negative	55	19	28		111	93		42	2	58		158	46		56	23	23		102	102	
Ulcer																					
positive	19	6	9	0.972	37	31	0.994	11	2	21	0.204	53	15	0.664	20	6	8	0.778	36	32	0.671
negative	60	21	31		122	102		42	1	69		180	44		60	26	26		112	112	
Hematuria																					
positive	37	15	21	0.687	79	67	0.906	24	2	47	0.615	118	28	0.662	39	15	19	0.724	77	69	0.482
negative	42	12	19		80	66		29	1	43		115	31		41	17	15		71	75	
Proteinuria																					
positive	46	13	23	0.647	92	72	0.523	23	2	57	0.04	137	27	0.071	48	17	17	0.57	82	82	0.791
negative	33	14	17		67	61		30	1	33		96	32		32	15	17		66	62	
Hypocomplemetemia																					
positive	70	21	31	0.187	132	112	0.784	45	2	75	0.563	195	49	0.906	71	25	26	0.174	123	121	0.832
negative	9	6	9		27	21		8	1	15		38	10		9	7	8		25	23	
Thrombocytopenia																					
positive	26	10	12	0.835	50	46	0.529	22	0	26	0.148	74	22	0.419	28	9	11	0.781	27	25	0.844
negative	53	17	28		109	87		31	3	64		159	37		52	23	23		121	119	

SLE is one of the most common autoimmune diseases in the world.^[7]^ Various genetic factors are associated with susceptibility to developing SLE. Different SNPs have been associated with SLE in several populations. Rheumatoid arthritis (RA) and SLE belong to rheumatic diseases. Vander Cruyssen shown the increased frequency of A allele of rs2989727 and G allele of rs1071583 in RA patients.^[8]^ We speculated that rs2989727 and rs1071583 SNP may be associated with susceptibility to SLE. However, the results were not as we had expected. This study suggested that SNP rs7851696, rs2989727 and rs1071583 of FCN gene might be not associated with genetic susceptibility to SLE. The inconsistence may relate to heterogeneity among different diseases, ethnicity, experimental and analytical methods, and sample size. Nevertheless, the relationship of ficolin concentration with SLE cannot be ruled out since its levels were not evaluated here.

In further analysis, we found that FCN gene SNPs were related to laboratory characteristics, the differences of the distribution of genotype of FCN rs7851696 SNP between proteinuria positive groups and proteinuria negative groups. Due to proteinuria being a common clinical manifestation of SLE and influenced by multiple factors, the proteinuria levels of the same patient may fluctuate over time. Therefore, the genetic differences observed between proteinuria positive and negative groups have limited significance. Therefore, more research is needed to confirm this performance. Moreover, one must address the limitations of our study. First, the sample number may be quantitively unsatisfactory to reveal final conclusions regarding genetic phenomena in SLE.^[9]^ Furthermore, this study was a single-center research.

Taking into account the shortcomings of this study, conclusions should be made with caution. We have not able to demonstrate that SLE susceptibility was related to the distribution of genotypes and alleles of FCN gene SNPs. However, the rs7851696 genotype may be associated with clinical features of SLE. The further studies are needed to reveal molecular genetics research of SLE.
